# The possible benefits of reduced errors in the motor skills acquisition of children

**DOI:** 10.1186/1758-2555-4-1

**Published:** 2012-01-09

**Authors:** Catherine M Capio, Cindy HP Sit, Bruce Abernethy, Rich SW Masters

**Affiliations:** 1 Institute of Human Performance, University of Hong Kong Pokfulam Road, Hong Kong, Hong Kong SAR; 2 Faculty of Kinesiology and Rehabilitation Sciences, Katholieke Universiteit Leuven Tervusevestraat, Heverleee 3001, Belgium; 3 Department of Sports Science and Physical Education, Chinese University of Hong Kong Kwok Sports Building, Shatin NT, Hong Kong SAR; 4 School of Human Movement Studies, University of Queensland Blair Drive, Brisbane Queensland 4072, Australia

**Keywords:** Motor learning, Children, Rehabilitation

## Abstract

An implicit approach to motor learning suggests that relatively complex movement skills may be better acquired in environments that constrain errors during the initial stages of practice. This current concept paper proposes that reducing the number of errors committed during motor learning leads to stable performance when attention demands are increased by concurrent cognitive tasks. While it appears that this approach to practice may be beneficial for motor learning, further studies are needed to both confirm this advantage and better understand the underlying mechanisms. An approach involving error minimization during early learning may have important applications in paediatric rehabilitation.

## Background

Motor learning is the process of acquiring movement skills [1]. Conventional (explicit) theories posit that motor skills are initially learned explicitly through cognitive processes that generate declarative knowledge [2]. Such knowledge is made up of information that learners can describe verbally [3], and includes rules for the execution of the desired movement [4]. With increasing proficiency, movement skills become automated and performance becomes implicit, such that the declarative knowledge becomes inaccessible or unnecessary for movement control [5]. Masters [6] developed an alternative, implicit motor learning approach, in which movements are acquired *without *early dependence on working memory; thereby possibly bypassing the declarative stage that is characteristic of early explicit learning [7].

While there is considerable evidence to support the efficacy of an implicit motor learning approach, the bulk of the evidence comes from studies of adults. There is little evidence derived from children, whose information processing and cognitive abilities are still undergoing maturation [8]. As a consequence, the generalisability of implicit motor learning principles to children is unclear [9]. An understanding of children's movement skills learning is particularly important because engagement in motor activity is a prerequisite for development of the motor skills that are fundamental to functional tasks, school participation, and games and recreation [8] in later years of life. This paper aims to briefly review evidence from recent studies that have examined implicit motor learning in children using an errorless learning paradigm [10,11] and to explore the theoretical basis of implicit motor learning in children.

## Errors in motor learning

Whether the experience of errors during motor learning is a desirable component or not is a subject of debate. One view is that skill learning benefits from mistakes [12], whereas another view is that the formation of correct motor programs is delayed by the production of errors [13]. Recent research suggests that reducing errors during the early stages of motor learning is beneficial when the task involves complex, functional skills, such as skiing [14] or golf putting [4].

Maxwell and colleagues [4] proposed that errorless learning paradigms should constrain the environment to minimize the amount of errors that are committed, thereby reducing the need to test alternative movement solutions to correct errors. They showed that errorless learning resulted in motor learning that was largely implicit or non-conscious, with low accrual, or awareness, of declarative knowledge about the skill. Task performance was also found to be robust in the presence of a secondary cognitive task. Subsequent studies have shown that reduction of errors in the early stages of motor learning resulted in skills that were stable against physiological fatigue, were retained longer [15], and yielded superior performance [16].

## Errorless learning paradigm in children

The decreased cognitive demands associated with reduced commission of errors, suggests that children, whose cognitive resources are still in development, may gain particular benefit from an errorless approach to motor learning. Maxwell, Masters and Hammond [11] first explored this possibility in a study involving children who learned golf putting in either an errorless or error-strewn learning environment. For children in errorless learning conditions, golf putting distances were incrementally increased such that the participants began with an easy task, which gradually became more difficult. In contrast, children in error-strewn conditions practiced initially with a difficult task (far distance), which was incrementally decreased. Skills learnt via errorless learning were found to be unaffected by performing a concurrent cognitive task that demanded additional attention resources. Maxwell et al. [11] also classified the children as either having high or low motor abilities, and their study provided some evidence to suggest that reducing practice errors was beneficial particularly for children with lower motor abilities.

As is characteristic of many motor learning studies, the initial evidence supporting the errorless learning approach in motor learning for children was based on a laboratory experiment. Motivated by the need for a more applied investigation, a field-based study by Capio et al. [10] utilized this learning paradigm to examine the learning of a fundamental movement skill by children in a primary school setting. Overhand throwing was practised in either an errorless or error-strewn learning environment, within the context of physical education lessons. Children who learned overhand throwing with an approach that generated fewer errors were found to achieve superior movement patterns and throwing accuracy relative to those who practised in an error-strewn environment. Additionally, children who learned overhand throwing with few errors showed stable performance while engaged in a secondary cognitive task of counting backwards. This observation was consistent with findings of implicit motor learning investigations in adults [2]. Similar to the initial study by Maxwell et al. [11], the children were also grouped according to their abilities prior to practice (high, medium, low ability). Again, those in the low ability group were found to benefit most from the errorless learning approach. This field-based study therefore confirmed that the errorless learning paradigm is beneficial as well as feasible in a school setting.

Children of lower abilities, such as those with intellectual disabilities (ID), also appear to benefit from motor learning with reduced practice errors. Children with ID have less proficient movement skills, associated with their impaired cognitive processing abilities [17]. In a recent study [18] overhand throwing practice was incorporated in the adapted PE lessons of children with mild intellectual disability. Results showed that while all participants achieved gains in throwing movement pattern and throwing accuracy, those who practised in an errorless learning environment were found to have greater improvements than those who practised in an error-strewn condition. Moreover, those children with ID who experienced fewer practice errors were capable of effective overhand throwing while performing a secondary cognitive task.

## Discussion

Motor learning approaches for children need to accommodate their evolving cognitive abilities. Younger children have been found to approach information processing differently, such that they tend to rely more on visual codes initially and learn to use verbal labels as they get older [19]. Moreover, while the initial declarative stage that is characteristic of explicit motor learning has an associated verbal monitoring process, language ability develops relatively later than movement skills like walking [20]. As the implicit motor learning approach purports to skip the initial declarative stage of learning, developmental changes may partially explain why this approach seems to benefit children. It appears that by reducing the number of practice errors in the early stages of learning, cognitive processing load during movement skills performance is reduced such that the acquired skills are less susceptible to disruption from secondary cognitive tasks [10]. However, it must be noted that further verification is needed to establish whether the skills learnt in the errorless learning paradigm are indeed implicit. Nevertheless, despite the absence of a measure of declarative knowledge accumulation, the apparent benefits associated with errorless learning environments may provide an important basis for further work in implicit motor learning in children.

Reber [21] used an evolutionary framework to argue that implicit learning processes constitute an older cognitive system than explicit learning processes. Consequently, implicit learning should be largely unaffected by either age or intelligence (IQ). Such an evolutionary perspective offers an explanation for why the patterns observed in implicit learning among adult learners have been replicated in children despite different cognitive processing abilities. Consistent with the findings of the recent studies [10,11,18], a lack of sensitivity to both age [22] and IQ [23] has also previously been demonstrated for implicit motor learning in simpler sensorimotor tasks.

Alternative motor learning perspectives may also explain the apparent effectiveness of the errorless learning approach in children. Developmental principles of motor development have been influenced by the dynamical systems perspective [24], for example, with movement viewed as a consequence of dynamic interactions between internal (e.g., neurological structure) and external constraints (e.g., feedback from the environment). If the environmental constraints are consistent, stable movement patterns develop [25]. It may be that in restricting practice errors, environmental constraints become more consistent, which facilitates stable movement patterns. What remains to be determined, however, is whether, and to what degree, emphasizing errorless learning may restrict the opportunities to fully explore the boundary conditions under which different movement patterns can (and cannot) be used successfully. This aspect relates to the potential limitation in the development of movement adaptability.

The ecological perspective on motor skills development in children emphasizes the role of the environment in promoting developmental change [26], and in the eventual development of effective movement skills. While the environmental constraints that are manipulated in motor learning strategies are often physical in nature (e.g., equipment, setting), social aspects of the environment are particularly relevant for young learners whose performances are strongly influenced by their peers [27]. For instance, preschool children who underwent training in object control skills were found to have improved perceptions of their physical competence when the learning environment accommodated their desired levels of difficulty [28,29]. It has been suggested that the opportunities children have to experience success, contribute to their early self-judgment of their abilities and capabilities [30,31], encouraging subsequent performance of the learned skills. This socio-affective aspect of motor skills acquisition may also be a factor that leads to the possible benefits of errorless learning in children.

## Implications

Rehabilitation programs for children include multi-disciplinary approaches that foster capacities and social participation [32]. In the framework of the International Classification of Function, Disability, and Health (ICF), a holistic perspective on a child's status includes not only the movement performance itself, but also the context in which it is applied in the real world (e.g., school, playground) [33]. Such considerations raise issues related to diverse and multiple stimuli and performance pressures that are present in a child's daily environment. Movement skills that will remain stable in the face of such challenges in the environment are thus desirable for children. A reduction of practice errors during learning may therefore be useful in planning rehabilitation and sports programs for children.

The findings that were summarized in this paper also signal the need to conduct further research to establish theoretical and practical evidence. For instance, future work is needed to examine if benefits persist across different developmental conditions such as cerebral palsy, Down syndrome, or developmental coordination disorder. Furthermore, the impact of an errorless learning approach on movement adaptability (as opposed to consistency and stability) has yet to be examined. Ultimately, the mechanisms underpinning the effects of errorless learning in motor skills acquisition in children clearly require further exploration and understanding.

## Competing interests

The authors declare that they have no competing interests.

## Authors' contributions

CMC developed the main structure of the current concept paper and carried out two of the studies that were focal reference points in the manuscript. CHPS conceived of the current concept paper and contributed to the structure and content of the manuscript. BA contributed to structuring and refining the critical points of argument of the current concept. RSWM developed the main concept being discussed through numerous studies, and participated in all the key reference studies of the current concept. All the authors contributed to the writing, and have read and approved the final version of the manuscript.
